# Estimation of soybean yield based on high-throughput phenotyping and machine learning

**DOI:** 10.3389/fpls.2024.1395760

**Published:** 2024-06-06

**Authors:** Xiuni Li, Menggen Chen, Shuyuan He, Xiangyao Xu, Lingxiao He, Li Wang, Yang Gao, Fenda Tang, Tao Gong, Wenyan Wang, Mei Xu, Chunyan Liu, Liang Yu, Weiguo Liu, Wenyu Yang

**Affiliations:** ^1^ College of Agronomy, Sichuan Agricultural University, Chengdu, China; ^2^ Sichuan Engineering Research Center for Crop Strip Intercropping System, Chengdu, China; ^3^ Key Laboratory of Crop Ecophysiology and Farming System in Southwest, Ministry of Agriculture, Chengdu, China

**Keywords:** RGB, soybean, yield, machine learning, estimation

## Abstract

**Introduction:**

Soybeans are an important crop used for food, oil, and feed. However, China’s soybean self-sufficiency is highly inadequate, with an annual import volume exceeding 80%. RGB cameras serve as powerful tools for estimating crop yield, and machine learning is a practical method based on various features, providing improved yield predictions. However, selecting different input parameters and models, specifically optimal features and model effects, significantly influences soybean yield prediction.

**Methods:**

This study used an RGB camera to capture soybean canopy images from both the side and top perspectives during the R6 stage (pod filling stage) for 240 soybean varieties (a natural population formed by four provinces in China: Sichuan, Yunnan, Chongqing, and Guizhou). From these images, the morphological, color, and textural features of the soybeans were extracted. Subsequently, feature selection was performed on the image parameters using a Pearson correlation coefficient threshold ≥0.5. Five machine learning methods, namely, CatBoost, LightGBM, RF, GBDT, and MLP, were employed to establish soybean yield estimation models based on the individual and combined image parameters from the two perspectives extracted from RGB images.

**Results:**

(1) GBDT is the optimal model for predicting soybean yield, with a test set R2 value of 0.82, an RMSE of 1.99 g/plant, and an MAE of 3.12%. (2) The fusion of multiangle and multitype indicators is conducive to improving soybean yield prediction accuracy.

**Conclusion:**

Therefore, this combination of parameters extracted from RGB images via machine learning has great potential for estimating soybean yield, providing a theoretical basis and technical support for accelerating the soybean breeding process.

## 1 Introduction

Soybeans, a crucial oilseed economic crop, serve as a significant source of plant protein and fat in human diets. In addition, soybeans are second only to cash food crops such as wheat, rice and corn, and their trade is the largest among all agricultural products (Zhou and Yang, 2021). In recent decades, China has faced severe soybean self-sufficiency. According to statistics from the U.S. Department of Agriculture, China’s annual soybean imports exceed 100 million tons, with an average yield per unit area of only 132.4 kg/mu, which is significantly lower than the global average of 188.7 kg/mu. The economic returns from soybean cultivation are notably lower than those from summer crops such as corn, leading to a lack of enthusiasm among farmers for soybean cultivation (Ren et al., 2021). This situation perpetuates a negative cycle for soybean cultivation, emphasizing the importance of developing high-yield soybean varieties. Yield monitoring is a crucial parameter for assessing soybean productivity during the harvesting process. Traditional yield survey methods rely on the experience of farmers or professionals, primarily through destructive sampling, which is time-consuming, labor-intensive, and inherently uncertain. Therefore, nondestructive and accurate yield monitoring is highly important for soybean production. Yield is a comprehensive indicator influenced by factors such as genotype, the environment, and their interactions, making yield estimation highly challenging. To promote efficient soybean breeding, the following question arises: how can soybean yield be accurately and efficiently predicted?

In precision agriculture, a recent hotspot, nondestructive estimation technologies have been developed, providing new methods and means for crop growth estimation and demonstrating promising applications in crop yield estimation. Previous studies have shown that images captured by sensors such as RGB cameras (Ji et al., 2022), thermal infrared cameras (4), hyperspectral cameras (Chiozza et al., 2021), and computed tomography (CT) scanners (Hughes et al., 2017) can be used to extract multiple image traits. These traits can be used to establish predictive models for estimating crop yield. Among these sensors, thermal infrared (TIR) cameras operating under field conditions are strongly influenced by environmental temperature (Hu et al., 2017) and have very low resolution. Hyperspectral cameras, with multiple continuous bands, can acquire spectral images in various bands. However, due to the large amount of information, imaging is time-consuming, and image processing is slow. However, CT scanners are expensive and challenging to operate. In comparison, RGB cameras, as image acquisition devices, have the advantages of low information acquisition costs, small size, high resolution, and simple operation. These scanners have been widely used in crop monitoring (Yamaguchi et al., 2020). RGB images can record brightness values (DNs) of the red, green, and blue bands and, based on this, undergo color space conversion to calculate vegetation indices. Compared to spectral images or multisource data fusion, RGB images are correlated with a small amount of data and are easy to handle. Therefore, over the past decade, efforts have been made to develop the application of RGB cameras in crop yield estimation.

Machine learning (ML) is a significant branch of computer science, and with the continuous advancement of sensor technology and image-processing techniques, ML has found extensive applications in various aspects of precision agriculture research, including yield estimation. In 2021, Saul Justin Newman et al. demonstrated the potential of ML algorithms for robustly predicting important agronomic traits, including yield, and developing and testing new interpretable models in crop biology (9). As an emerging and more complex statistical model, ML can better describe the nonlinear relationship between input variables and predicted outcomes. Recent studies have consistently shown the significant advantages of this approach over linear models in yield prediction (Cao et al., 2022). ML algorithms have been widely used to establish predictive models relating image features to biological parameters. ML algorithms exhibit greater accuracy and efficiency than simple linear regression models (Wang et al., 2021).

Various methods have been proposed and applied to estimate crop yield (Li et al., 2021). employed the random forest method to achieve dynamic yield prediction for three crops in China—winter wheat, corn, and rice. They explored the optimal lead time for yield prediction for different crops and assessed the importance of various predictive factors. Minghan Cheng et al. conducted research on a prediction algorithm for Chinese corn yield using two machine learning methods, random forest regression (RFR) and gradient boosting decision trees (GBDTs). Their results showed that earlier lead times resulted in lower prediction accuracy, but the accuracy remained relatively high within at least 24 days before maturity (coefficient of determination (R^2^)>0.77, relative root mean square error (rRMSE)<16.92%) (Cheng et al., 2022, Jin et al., 2017). developed a method for winter wheat yield estimation by combining the AquaCrop model with optical and radar imaging data using a location and orientation system algorithm, which showed a high correlation between the predicted and measured yields (Gilliot et al., 2020). demonstrated the potential of predicting corn yield based on extracted plant height from images (Feng et al., 2020). used ensemble machine learning models for in-season alfalfa yield estimation (Sun et al., 2020). developed six mainstream machine learning models to estimate potato tuber yield and obtained satisfactory results. Maitiniyazi Maimaitijiang et al (Cao et al., 2022). demonstrated that using low-cost drones for multimodal data fusion under a deep neural network (DNN) framework can provide relatively accurate and robust soybean yield estimates. However, the predictive performance of models varies for different crops and environmental parameters, and limited research has explored the effects of different machine learning models on the prediction of individual soybean plant yields for multiple varieties.

The gradient boosting decision tree (GBDT) model is an additive model and a form of boosting in ensemble learning (Shi et al., 2018). This model reduces the residuals during training by continually combining linear combinations of functions to achieve regression. The light gradient boosting machine (LightGBM) is another popular gradient boosting method known for reducing errors, thereby improving accuracy and speed. However, this approach does not support string-type data and requires special algorithms for splitting categorical data, as it requires integer values (such as indices) instead of column string names (Brinkhoff et al., 2023; Seireg et al., 2023). The categorical boosting (CatBoost) algorithm, an open-source machine learning library released by the Russian search giant Yandex in 2017, is also part of the boosting algorithm series. CatBoost is a novel machine learning algorithm framework based on the gradient boosting decision tree (GBDT). In contrast to LightGBM, CatBoost can automatically convert strings into index values and handle missing numerical values. Unlike traditional neural network models, CatBoost does not require many samples for training; it adapts well to training with small-scale samples and provides high-precision diagnostics. The advantages of CatBoost include overcoming gradient bias, effectively addressing prediction bias, improving algorithm accuracy, enhancing generalizability, and preventing overfitting (Huang, 2020; Liu, 2020; Lu et al., 2022).

The random forest (RF) algorithm was proposed by Breiman in 2001 and is an ensemble machine learning method based on multiple classification regression trees (Breiman, 2001). The basic concept involves creating homogeneous subsets through bootstrapping and growing decision trees in each subset (number of trees: ntree) from the training dataset. The final result of RF regression is obtained by averaging all the decision trees (Breiman, 2001). Due to repeated sampling, RF regression can effectively reduce overfitting (Breiman, 2001).

The multilayer perceptron (MLP) model is a simple neural network and one of the earliest models in artificial intelligence. This model is the most widely used artificial intelligence model in all scientific numerical modeling fields (Paswan and Begum, 2013; Khalifani et al., 2022). MLPs typically include a set of sensory units (basic neurons) and consist of an input layer, one to several hidden layers, and an output layer. This method creates nonlinear mappings between input target samples, and input signals from the input layer to the output layer propagate forward (Aghelpour and Varshavian, 2021).

The development of nondestructive estimation techniques enables efficient and accurate monitoring of soybean yield, significantly shortening the time required for soybean breeding, meeting the needs of breeders, and facilitating efficient breeding. Therefore, the purpose of this study was to evaluate the accuracy of soybean yield estimation using five machine learning algorithms (CatBoost, LightGBM, RF, GBDT, and MLP) and to determine the optimal model for early soybean yield estimation using phenotypic features extracted from multiangle RGB images. This approach aims to increase the efficiency of obtaining soybean biological traits and accelerate the soybean breeding process.

## 2 Materials and methods

### 2.1 Experimental site overview

**Figure 1:**
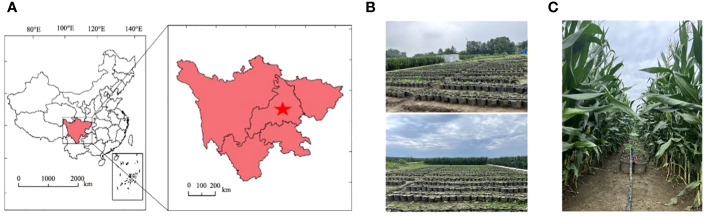
Study area and experimental design. **(A)** Geographic location of the Sichuan Agricultural University Modern Agriculture Research and Development Base; **(B)** sole cropping of soybeans; and **(C)** strip intercropping of corn and soybeans.

The experiment was conducted during 2022–2023 at the Chongzhou Experimental Base of Sichuan Agricultural University (103°39’E, 30°33’N), as depicted in **Figure 1A**. This region has a subtropical monsoon climate, with an average temperature of 16.2°C, an annual total sunshine duration of 1400 hours, and an annual total rainfall of 918 mm. The basic chemical properties of the 0–20 cm soil layer at the experimental site were as follows: organic matter content, 24.3 g·kg^-1^; total potassium, 15.2 g·kg^-1^; total nitrogen, 1.6 g·kg^-1^; total phosphorus, 1.3 g·kg^-1^; available potassium, 169.4 mg·kg^-1^; available nitrogen, 299.5 mg·kg^-1^; and available phosphorus, 36.5 mg·kg^-1^.

### 2.2 Experimental design

The experimental materials consisted of 240 soybean varieties (a natural population formed by four provinces in China: Sichuan, Yunnan, Chongqing, and Guizhou) (**Appendix 3**). As shown in **Supplementary Figure S1**, the genetic diversity of this population is extremely extensive, and all of these populations were planted in 2022. In 2023, based on the previous year’s yield, extremely low-performing varieties were excluded, resulting in the final cultivation of 202 soybean varieties. Over the two years of field experiments, each variety was subjected to three replications and two planting methods to increase yield differences (sole cropping and strip intercropping with corn). In the strip intercropping system with corn, the corn variety used was Zhongyu 3, a semicompact spring corn. The planting materials used were provided by the Crop Strip Composite Planting Engineering Technology Research Center of the College of Agronomy, Sichuan Agricultural University. The field layout is shown in **Figures 1B, C**. In the corn–soybean strip intercropping system, two rows of corn (corn strip) were intercropped with two rows of soybean (soybean strip), with a length of 20 m and a width of 2 m (the row spacing for both the corn-corn and soybean-soybean systems was 40 cm, and the spacing between the corn and soybean strips was 60 cm). Both corn and soybean were single-hole planted, with a hole spacing of 20 cm for corn. For soybean, pots were planted with a diameter of 25 cm, a diameter of 20 cm, and a height of 25 cm; the pots were filled with 10 kg of soil. Under the intercropping treatment, the potted soybeans were placed in wide rows of corn, with two pots placed side by side in each strip. The soybean plant density and row spacing under the monocropping treatment were consistent with those under the intercropping treatment. The base fertilizer for corn was compound fertilizer (N:P:K = 13:5:7) applied at a rate of 923 kg·hm^-2^. At the jointing and tasseling stages, urea (N ≥ 46%) was applied at rates of 98 kg·hm^-2^ and 163 kg·hm^-2^, respectively. No fertilizer was applied throughout the entire growth period of the soybeans.

### 2.3 High-throughput phenotypic data acquisition

**Figure 2:**
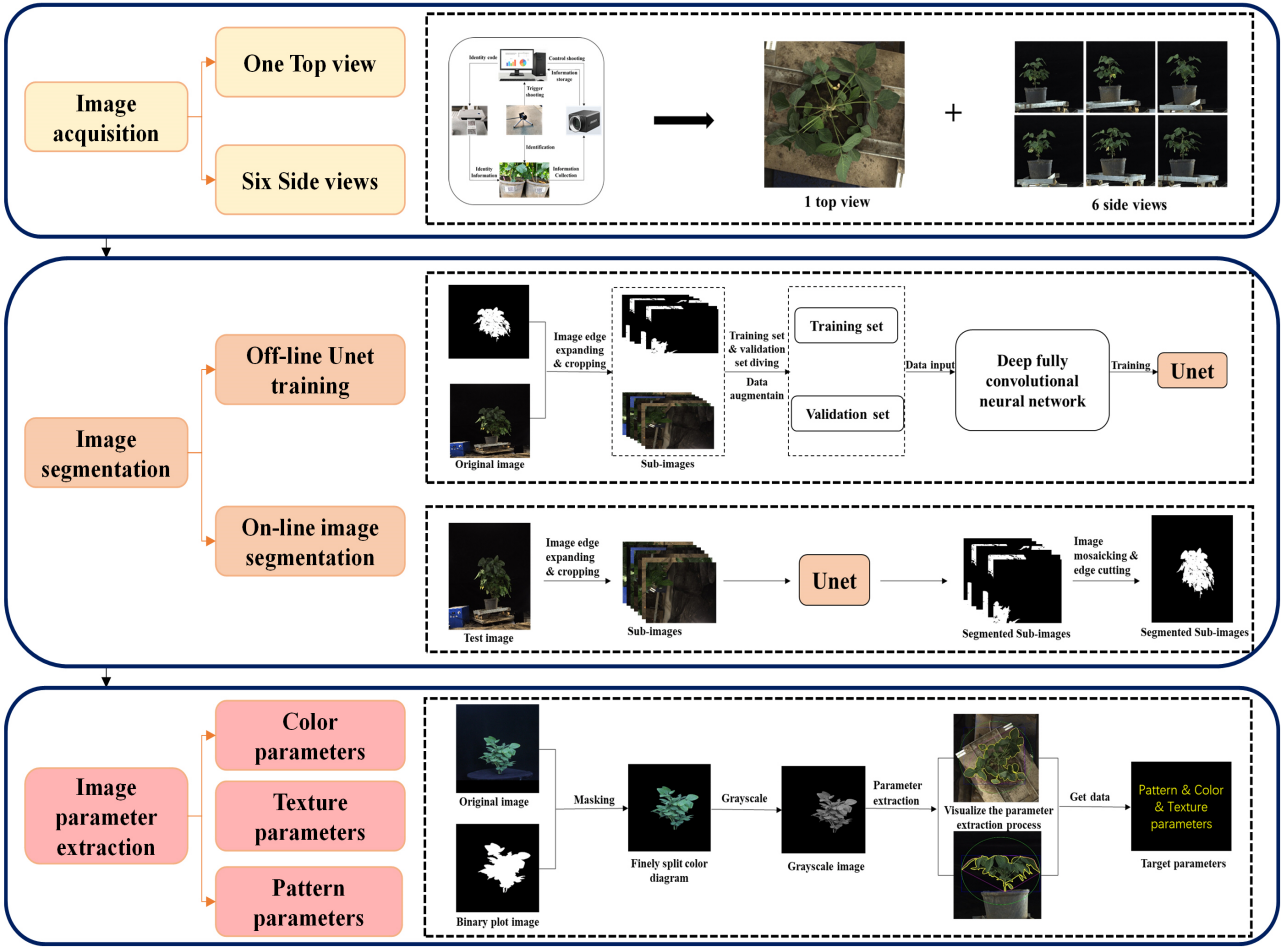
High-throughput phenotyping of soybeans.

The flowchart depicted in **Figure 2** illustrates the methodology employed in this study for obtaining high-throughput soybean phenotypic data. This process included image acquisition, image segmentation (offline model training and online image segmentation), and parameter extraction. The details of this flowchart are discussed below. In this study, a total of three categories of parameters were extracted from two shooting angles, including 6 color parameters each for the top and side views, 17 textural parameters each for the top and side views, and 16 and 30 morphological parameters for the top and side views, respectively (refer to **Appendix 1**). In this research, the color, texture, and morphological parameters from the top and side views were individually or collectively used to estimate soybean yield.

#### 2.3.1 Image acquisition

In 2022–2023, we utilized a self-developed single soybean plant imaging platform from Sichuan Agricultural University as the capture platform. This platform, centered on an automatic rotating table, is equipped with industrial cameras on both the top and side of the table. The table rotation speed and number of cycles are controlled by a programmable logic controller (PLC). The selected Hikvision industrial cameras (MV-CH250–90GC, China) paired with Hikvision robot lenses (MVL-KF1624M-25MP, focal length 16 mm, maximum aperture F2.4, 1.2 C, Hangzhou, China) were used as sensors for capturing images. During the capture process, the camera parameters were set as follows: focal lengths for the side and top cameras were 2.3 mm and 2.4 mm, respectively; shooting distances were 2.6 m and 1.8 m, respectively; and the camera mode was set to aperture priority (AV) with an aperture size of 2.4 and automatic white balance, ensuring a camera frame rate ≥4.5 fps. The side and top images were stored in JPG format with resolutions of 4000*4000 and 4604*4604, respectively. A white standard board with a diameter of 30 cm was used as the scale, and a scale image of both the top and side views was collected to calculate the values of the extracted image features.

RGB images were collected during the R6 stage (pod filling stage) of soybeans. Each time, images were captured by randomly selecting 3 pots from each treatment. The soybean plants were placed on the turntable, and every 60° rotation, a side image was obtained. Additionally, a random overhead view was captured. In total, one overhead view and six side views were collected for each soybean plant. A total of 18,564 images were captured, 15,912 of which were collected in 2022 (8,640 side views and 1,440 top views), and 8,484 were captured in 2023 (7,272 side views and 1,212 top views). The specific imaging process is illustrated in **Figure 2**.

#### 2.3.2 Image segmentation

The soybean plant segmentation model utilized the U-Net neural network. In this study, 2000 images were used as the training set to train U-Net. The images were divided into training, testing, and validation sets at a ratio of 8:1:1. The U-net network was pretrained using the VOC 2012 dataset to enhance its feature extraction capability, and the obtained weights were utilized for model training. Subsequently, transfer learning was performed using the pretrained weights to conduct formal training on U-Net. The training was conducted on a computer with a Windows 10 system using a resolution of 2048×2048 and an NVIDIA GeForce RTX 3090 with 48 GB of memory. The training was implemented based on the PyTorch framework. The training process comprised two stages: the first stage involved freezing the main feature extraction network and enhancing the weights of the feature extraction network, training only the classification network. The parameters were adjusted as follows: learning rate = 110^-4, epochs = 60, batch size = 4, and learning rate decay = 0.92. In the second stage, the feature extraction network was unfrozen, and the entire network was trained. The parameters were adjusted as follows: learning rate = 110^-5, epochs = 50, batch size = 1, and learning rate decay = 0.95.

#### 2.3.3 Image parameter extraction

##### 2.3.3.1 Color features

Extraction was performed on the three channels (R, G, and B) of the finely segmented color image to calculate color trait values. The color parameters extracted in this study included the blue, green, red, blue-green, blue-red, and green-red ratios, comprising six indicators. The calculation formulas are as follows Equations (1–3):


(1) $$R\;=\;\frac{\sum_{i=1}^{n}R_{i}}{n}$$

(2) $$G\;=\;\frac{\sum_{i=1}^{n}G_{i}}{n}$$

(3) $$B\;=\;\frac{\sum_{i=1}^{n}B_{i}}{n}$$

In the formulas, Ri represents the pixel value of the red channel for the i-th pixel of the plant, R denotes the red mean, Gi represents the pixel value of the green channel for the i-th pixel of the plant, G denotes the green mean, Bi represents the pixel value of the blue channel for the i-th pixel of the plant, and B denotes the blue mean. n represents the number of pixels in the plant.

For each image, a set of R, G, and B values can be obtained, and the values of the side color parameters for each plant are the averages of these values from six images. The blue-green ratio, blue-red ratio, and green-red ratio are then calculated as the ratios of the blue mean to the green mean, the blue mean to the red mean, and the green mean to the red mean, respectively.

##### 2.3.3.2 Textural features

Image parameter extraction was conducted using Python 3.7 (Python Software Foundation, https://www.python.org/) and the scikit-learn library v0.21.3. Textural processing is a common method for extracting information from digital images, and although it lacks a formal definition, an intuitive description of textural information captures its properties well. Textural computation involves two main approaches: spectral methods based on the properties of the Fourier spectrum and detection of the global periodicity of the image by identifying high-energy narrow peaks in the spectrum (Bharati et al., 2004). On the other hand, statistical methods can extract the directionality, roughness, and degree of order in images. Textural traits were calculated using the gray-level co-occurrence matrix (GLCM), which was initially proposed by Hong Jiguang (Hong, 1984). By combining the overall soybean textural gray-level division, the color space of the color image was converted to the HIS color space, with the I channel serving as the grayscale image. The specific calculation formula is as follows Equation (4):


(4) $$I\;=\;\frac{R+G+B}{3}$$

The element H(i, j) in the gray-level co-occurrence matrix is defined as the total number of pixels with a normalized grayscale of i in the grayscale image and a normalized gradient of j in the gradient image. The probability of having a grayscale value of i and a gradient value of j is calculated as follows Equation (5):


(5) $$p(i.j)=H(i,j)/\sum_{i=1}^{N_{f}}H(i,j)$$

where i = 0, 1, 2…, *Nf* represents the normalized maximum grayscale value and j = 0, 1, 2…; a total of 15 textural traits were obtained. In addition, two histogram traits were included in this study. The textural parameters on the side were the averages of six images. The specific calculation formulas are provided in **Table 1**.

**Table 1:** Formulas for Textural Parameter Calculations.

Number	Full name	Calculation formula
1	Small gradient advantage	$T1=\left[\sum_{\text{i}=1}^{16}\sum_{\text{j}=1}^{16}\frac{\text{H}\left(\text{i},\text{j}\right)}{\text{j}2}\right]/\left[\sum_{\text{i}=1}^{16}\sum_{\text{j}=1}^{16}\text{H}(\text{i},\text{j})\right]$
2	Large gradient advantage	$T2=\left[\sum_{\text{i}=1}^{16}\sum_{\text{j}=1}^{16}j^{2}\text{H}\left(\text{i},\text{j}\right)\right]/\left[\sum_{\text{i}=1}^{16}\sum_{\text{j}=1}^{16}\text{H}(\text{i},\text{j})\right]$
3	energy	$T3={\sum_{\text{i}=1}^{16}\sum_{\text{j}=1}^{16}\left[P\left(\text{i},\text{j}\right)\right]}^{2}$
4	The gradient distribution has inhomogeneity	$T4=\left\{\sum_{i=1}^{16}{\left[\sum_{i=1}^{16}H\left(i,j\right)\right]}^{2}\right\}/\left[\sum_{i=1}^{16}\sum_{j=1}^{16}H(i,j)\right]$
5	Gradient average	${\text{μ}}_{2}=\sum_{j=1}^{16}j*[\sum_{j=1}^{16}P\left(i,j\right)]$
6	Gradient entropy	$T5=-\left\{\sum_{j=1}^{16}\left[\sum_{i=1}^{16}P\left(i,j\right)\right]*\log\left[\sum_{i=1}^{16}P(i,j)\right]\right\}$
7	Grayscale entropy	$T6=-\left\{\sum_{i=1}^{16}\left[\sum_{j=1}^{16}P\left(i,j\right)\right]*\log\left[\sum_{j=1}^{16}P(i,j)\right]\right\}$
8	Mixed entropy	$T7=-\sum_{i=1}^{16}\sum_{j=1}^{16}P(i,j)*logP(i-j)$
9	Differential moment	$T8=\sum_{i=1}^{16}\sum_{j=1}^{16}P(i,j){\left(i-j\right)}^{2}$
10	Deficit moment	$T9=\sum_{i=1}^{16}\sum_{j=1}^{16}\frac{1}{1+{\left(i,j\right)}^{2}}P(i,j)$
11	Gradient standard deviation	$\sigma_{2}=\{\sum_{j=1}^{16}{\left(j-\mu_{2}\right)}^{2}[\sum_{i=1}^{16}P(i,j)]{\}}^{1/2}$
12	Correlation	$T10=\frac{1}{\sigma_{1}\sigma_{2}}\sum_{i}^{16}\sum_{j}^{16}(i-\mu_{1})(j-\mu_{2})P(i,j)$
13	Grayscale histogram variance	$S^{2}=\frac{\sum_{i=0}^{255}{\left(x_{i}-x^{-}\right)}^{2}}{255}$
14	Grayscale histogram entropy	$H=\sum_{i=0}^{255}P_{i}\log P_{i}$
15	The grayscale is unevenly distributed	$T11=\{\sum_{i=1}^{16}[\sum_{j=1}^{16}H(i,j){]}^{2}\}/[\sum_{i=1}^{16}\sum_{j=1}^{16}H(i,j)]$
16	Gray average	$\mu_{1}=\sum_{i=1}^{16}i*[\sum_{j=1}^{16}P(i,j)]$
17	Grayscale standard deviation	$\sigma_{1}=\{\sum_{i=1}^{16}{\left(i-\mu_{1}\right)}^{2}[\sum_{j=1}^{16}P(i,j)]{\}}^{1/2}$

##### 2.3.3.3 Morphological features

The number of binary image pixels was calculated, and the side data represent the average of six sideview images. Definitions for each indicator are provided in **Appendix 2**.

### 2.4 Yield data collection

**Figure 3:**
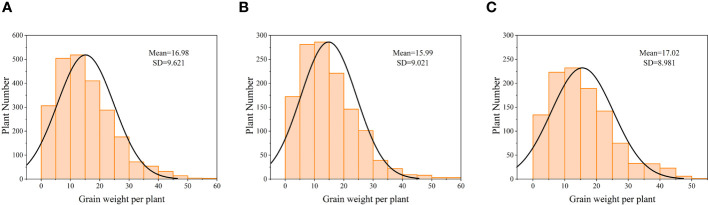
Normal distribution plot of manually measured individual soybean yields **(A)** 2381 trial materials. **(B)** 1291 training set materials. **(C)** 1091 test set materials.

After the soybeans matured, manual collection and recording of individual soybean yields were conducted. The yield distribution is depicted in **Figure 3**, and the data for both years follow a normal distribution.

### 2.5 Broad-sense heritability calculation

We evaluated the broad-sense heritability (*H^2^*) of the data over the two years using the formula *H^2^* = V_g_/(V_g_ + V_ll_/n + V_ly_/n + V_r_/n^2^). Here, V_g_ represents the genotypic variance, V_ll_ represents the variance between varieties and locations, V_ly_ represents the variance between varieties and years, and V_r_ represents the residual variance, with n denoting the number of replicates. Means and standard deviations were calculated using BLUP values, and the statistical significance of P values was determined using paired *t* tests.

### 2.6 Construction and evaluation of soybean yield estimation models

**Figure 4:**
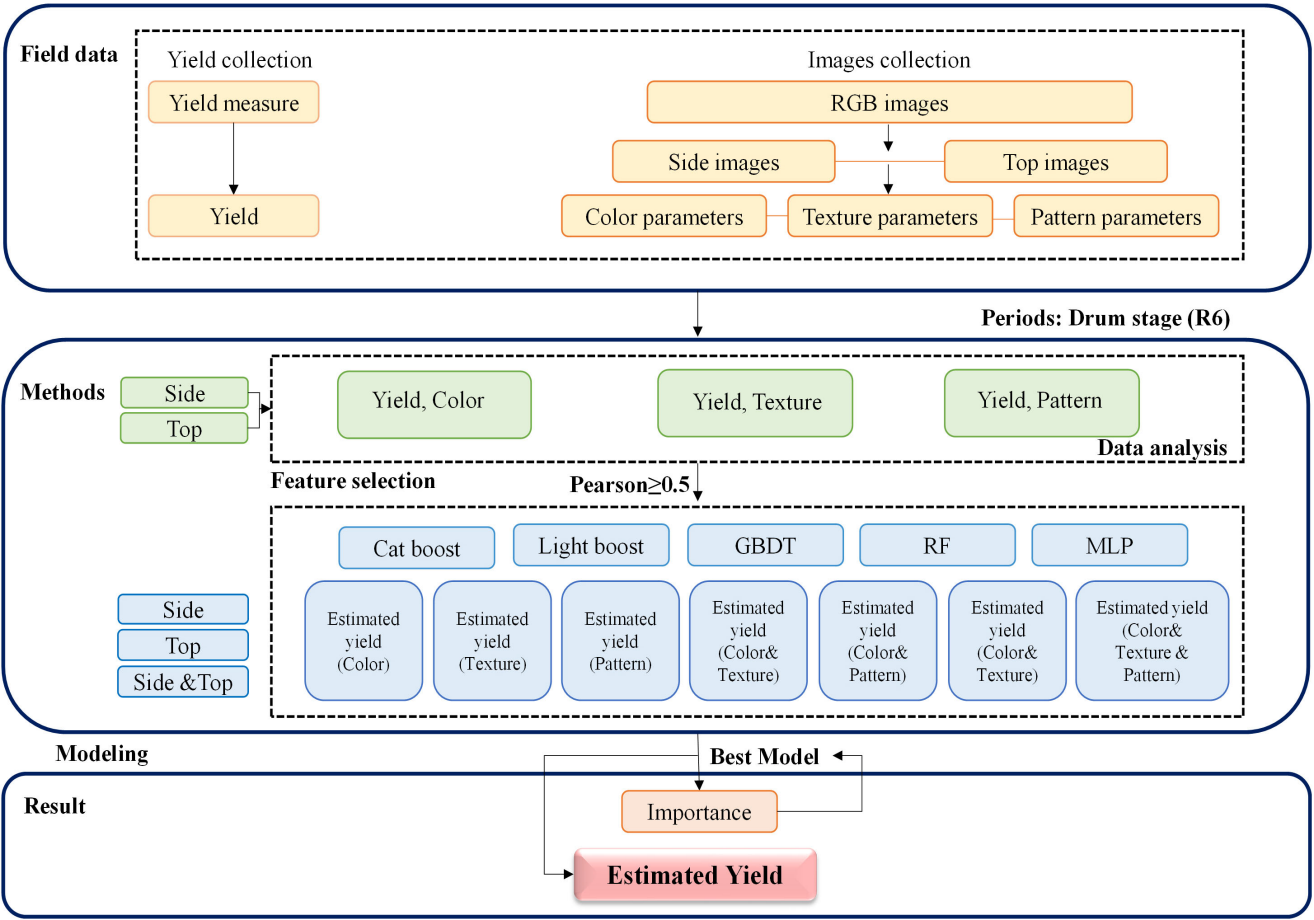
Data analysis workflow.

To further investigate the estimation accuracy of the yield prediction models, data preprocessing was performed using R Version 4.1.1 (R Foundation, Vienna, Austria). The outlier test function from the car package was utilized to remove outliers. A total of 149 outliers were eliminated from the 1440 datasets for 2022, and 121 outliers were removed from the 1212 datasets for 2023. Subsequent analyses were based on the remaining 2382 datasets. The statistical modeling was conducted using Python 3.7. In this study, five different regression methods were employed: CatBoost, LightGBM, RF, GBDT, and MLP. All the input parameters were normalized. The two years of data were divided into training and testing datasets based on the years. The samples from 2022, totaling 1291, were used as the training set, while those from 2023, totaling 1091, were used as the testing set. Tenfold cross-validation was also performed. All the numerical data were plotted using Origin 2019 and SPSS 2018. The data analysis methods employed in this study are illustrated in **Figure 4**.

#### 2.6.1 Machine learning parameter adjustment

In this study, the loss functions for all five models were set to ‘friedman_mse’, and a grid search was employed for hyperparameter tuning. The final settings for the GBDT model were a learning rate of 0.1, a maximum depth of 5, and a maximum iteration count of 800. For the CatBoost model, the final setting was a learning rate of 0. 05, a maximum tree depth of 10, and a maximum iteration count of 2000. In the LightGBM model, the final settings were a learning rate of 0.1 and a maximum tree depth of 10. In the RSF model, the optimal number of trees (ntree) for soybean yield estimation was determined by testing ntree values from 100 to 500 in increments of 50. The value 300 was chosen because it achieved stable and relatively low mean absolute error (MAE) and high R^2^ in the soybean yield estimation model. Other hyperparameters in the RF regression were set to the default values using the regressor function in the scikit-learn library. For the MLP model, the activation function was set to ‘relu,’ regularization coefficient to 0.01, two hidden layers were established with 100 neurons in the first layer and 50 neurons in the second layer, the learning_rate was set to ‘adaptive’, and the solver was set to ‘sgd.’

#### 2.6.2 Modeling evaluation

To reduce the impact of data partitioning on model estimation errors, the root mean square error (RMSE), MAE, and R^2^ were calculated to assess the performance of each estimation method. The specific calculation formulas are as follows Equation (6–8):


(6) $$RMSE=-\sqrt{\frac{1}{m}\sum_{i=1}^{m}{\left(yi-\hat{\text{yi}}\right)}^{2}}\;\;$$

(7) $$MAE=\frac{1}{m}\sum_{i=1}^{m}\left|\left(yi-\hat{\text{yi}}\right)\right|$$

(8) $$R^{2}=1-\frac{\sum_{1}^{i}{\left(\hat{\text{yi}}-yi\right)}^{2}}{\sum_{1}^{i}{\left(\bar{\text{yi}}-yi\right)}^{2}}\;$$

Here, $yi-\hat{\text{yi}}$ represents the difference between the actual values and predicted values on the test set.

## 3 Results

### 3.1 Correlations between color, texture, and morphological parameters and yield

**Figure 5:**
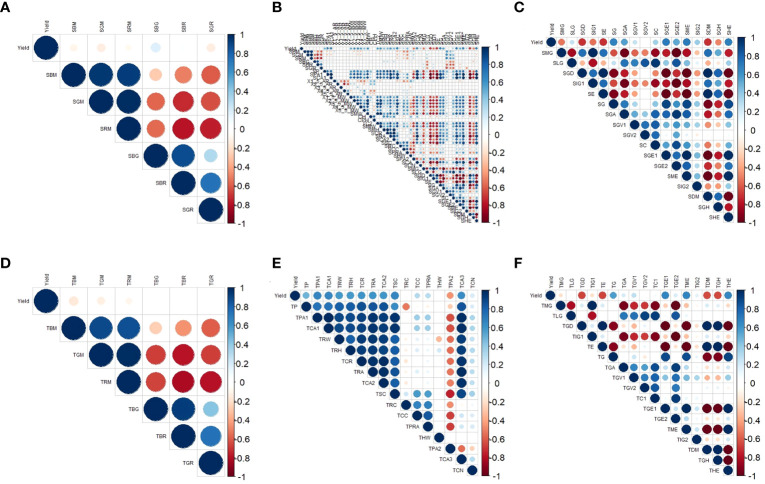
Correlation between three types of image parameters and yield. **(A)** Correlations between sideview color parameters and yield; **(B)** correlations between sideview morphology and yield; **(C)** correlations between sideview textural parameters and yield; **(D)** correlations between top-view color parameters and yield; **(E)** correlations between top-view morphology and yield; and **(F)** correlations between top-view textural parameters and yield.

The red area indicates a negative correlation, while the blue area indicates a positive correlation. Lighter colors represent weaker correlations. As shown in **Figure 5**, there were a total of 23 image indicators in the sideview that were absolute correlations (hereinafter referred to as correlations) with a yield greater than 0.5. Among the sideview color parameters, all the indicators were correlated with a yield less than 0.5 (**Figure 5A**). Among the sideview morphological parameters, 13 indicators had a correlation with a yield greater than 0.5, and except for SPA2 and SCA, the other 11 indicators were positively correlated with yield (**Figure 5B**), indicating that an increase in these 11 indicators leads to an increase in soybean yield. Among the sideview textural parameters, 10 indicators were correlated with a yield greater than 0.5. Among them, SGD, SIG1, SE, and SDM were negatively correlated with yield, while SG, SGA, SGE1, SGE2, SME, and SHE were positively correlated with yield (**Figure 5C**).

In the top view, a total of 16 image indicators were correlated with a yield greater than 0.5. According to the top-view color parameters, all the indicators were correlated with a yield less than 0.5 (**Figure 5D**). Among the top-view morphological parameters, 9 were correlated with a yield greater than 0.5, and all the indicators were positively correlated with yield (**Figure 5E**). Among the top-view textural parameters, 7 indicators exhibited a correlation with a yield greater than 0.5. Among them, TE, TDM, and TGH had negative correlations with yield, while TG, TGE1, TME, and THE had positive correlations with yield (**Figure 5F**).

An interesting phenomenon was observed here: the relationship between the image parameters and yield was quite similar between the side and top views. For example, SE and SDM exhibited a high negative correlation with yield, corresponding to TE and TDM, which also exhibited a high negative correlation with yield in the top-view image parameters. Similarly, SG, SGE1, SME, and SHE exhibited a high positive correlation with yield, corresponding to TG, TGE1, the TME, and the, which also showed a high positive correlation with yield in the top-view image parameters. Therefore, further exploration of the relationships between side- and top-view image parameters and soybean yield is particularly intriguing.

### 3.2 Prediction of yield based on sideview image parameters

The sideview image parameters with Pearson coefficients greater than 0.5 were selected for soybean yield estimation. In this study, the CatBoost, LightGBM, GBDT, RF, and MLP models were used to estimate soybean yield, and three combinations were trained and evaluated. The three combinations of input variables were named m1-m3, where m1 consists of morphological parameters (SPA1, SCA1, 1/5MW, 2/5MW, 3/5MW, 4/5MW, SMW, TC2, SMIW, SRA, SSC, SPA2, and SCA), m2 consists of textural parameters (SMG, SGD, SIG1, SE, SG, SGA, SGE1, SGE2, SME, and SDM), and m3 consists of morphological and textural parameters.

**Figure 6:**
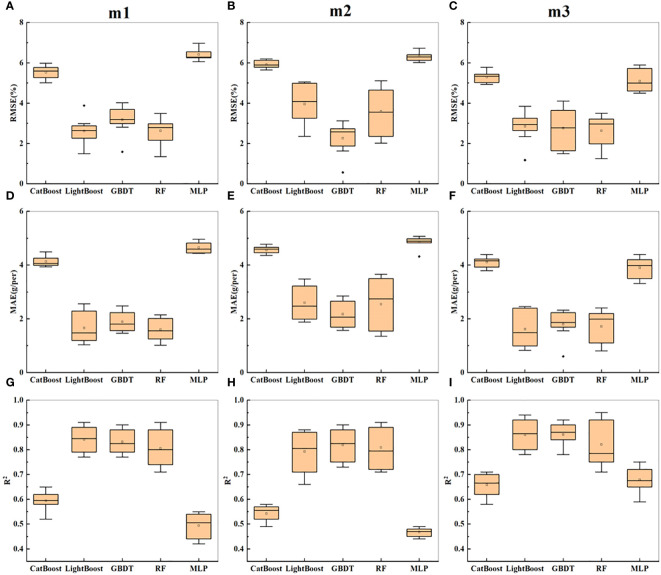
Presents the statistical results of yield prediction cross-validation based on side image parameters. **(A–C)** represent the RMSE of combinations m1-m3 across five machine learning models. **(D–F)** represent the MAE of combinations m1-m3 across five machine learning models. **(G–I)** represent the R^2^ of combinations m1-m3 across five machine learning models.

As shown in **Table 2**, the R^2^ value on the training set was generally greater than that on the test set, and the RMSE and MAE were generally lower than those on the test set. For m1, the prediction accuracy of the five models on the test set fluctuated greatly, with 0.42 ≤ R^2^ ≤ 0.77, and the GBDT and LightGBM models achieved the best prediction accuracy, both with R^2^ values of 0.77. The performance accuracy of RF was the lowest, and the prediction accuracies of CatBoost and MLP were the lowest (R^2^ ≤ 0.52), with the highest errors. For m2, the overall prediction accuracy of the five models was lower than that for m1, but the GBDT model still achieved the highest prediction accuracy, with R^2 =^ 0.73, while CatBoost and MLP yielded the lowest prediction accuracies, with R^2^ values of 0.49 and 0.44, respectively. For m3, after combining textural parameters with morphological parameters, the overall prediction accuracy of the five models slightly improved, with the MLP model showing the most significant improvement. According to **Figures 6A–I**, the error order is m2 > m1 > m3, and the R^2^ order is m3 > m1 > m2. With the enrichment of indicators, the prediction accuracy increased, and the error decreased, with morphological parameters leading to better estimation performance than textural parameters. Overall, in the prediction of soybean yield based on sideview image parameters, the optimal predictive model was GBDT.

**Table 2:** Prediction of yield based on sideview image parameters.

		m1			m2			m3		
		RMSE (%)	MAE (g/per)	R^2^	RMSE (%)	MAE (g/per)	R^2^	RMSE (%)	MAE (g/per)	R^2^
CatBoost	Val	5.18	3.93	0.65	5.72	4.36	0.58	4.93	3.79	0.71
	Cal	5.98	4.49	0.52	6.19	4.68	0.49	5.78	4.38	0.58
LightBoost	Val	1.49	1.03	0.91	2.56	1.88	0.88	1.17	0.82	0.94
	Cal	3.88	2.56	0.77	5.05	3.48	0.66	3.85	2.46	0.78
GBDT	Val	1.58	1.46	0.9	1.62	1.57	0.89	1.49	1.55	0.92
	Cal	4.02	2.48	0.77	3.12	2.85	0.73	4.10	2.32	**0.78**
RF	Val	1.35	1.02	0.91	2.01	1.35	0.91	1.24	0.81	0.95
	Cal	3.49	2.15	0.71	5.10	3.65	0.71	3.5	2.41	0.77
MLP	Val	6.05	4.44	0.54	6.40	4.83	0.49	4.49	3.31	0.75
	Cal	6.82	4.96	0.42	6.72	5.07	0.44	5.89	4.39	0.59

Val for the validation set, Cal for the test set. The bold font represents the optimal predicted values.

**Figure 6** shows the statistical results of cross-validation for soybean yield prediction based on side-image parameters. It can be seen that m3 still performed the best overall, with the highest prediction accuracy for the GBDT model, followed by LightBoost. However, compared to the prediction results in **Table 2**, the overall prediction accuracy improved.

### 3.3 Yield prediction based on top-image parameters

The soybean yield was estimated using top-image parameters with a correlation greater than 0.5. The three combinations of input variables were named M1-M3; M1 consisted of morphological parameters (TPA1, TCA1, TRW, TRH, TCR, TRA, TCA2, TSC, and TCA3), M2 consisted of textural parameters (TGD, TE, TG, TGE1, TME, TDM, and TGH), and M3 consisted of a combination of morphological and textural parameters.

**Figure 7:**
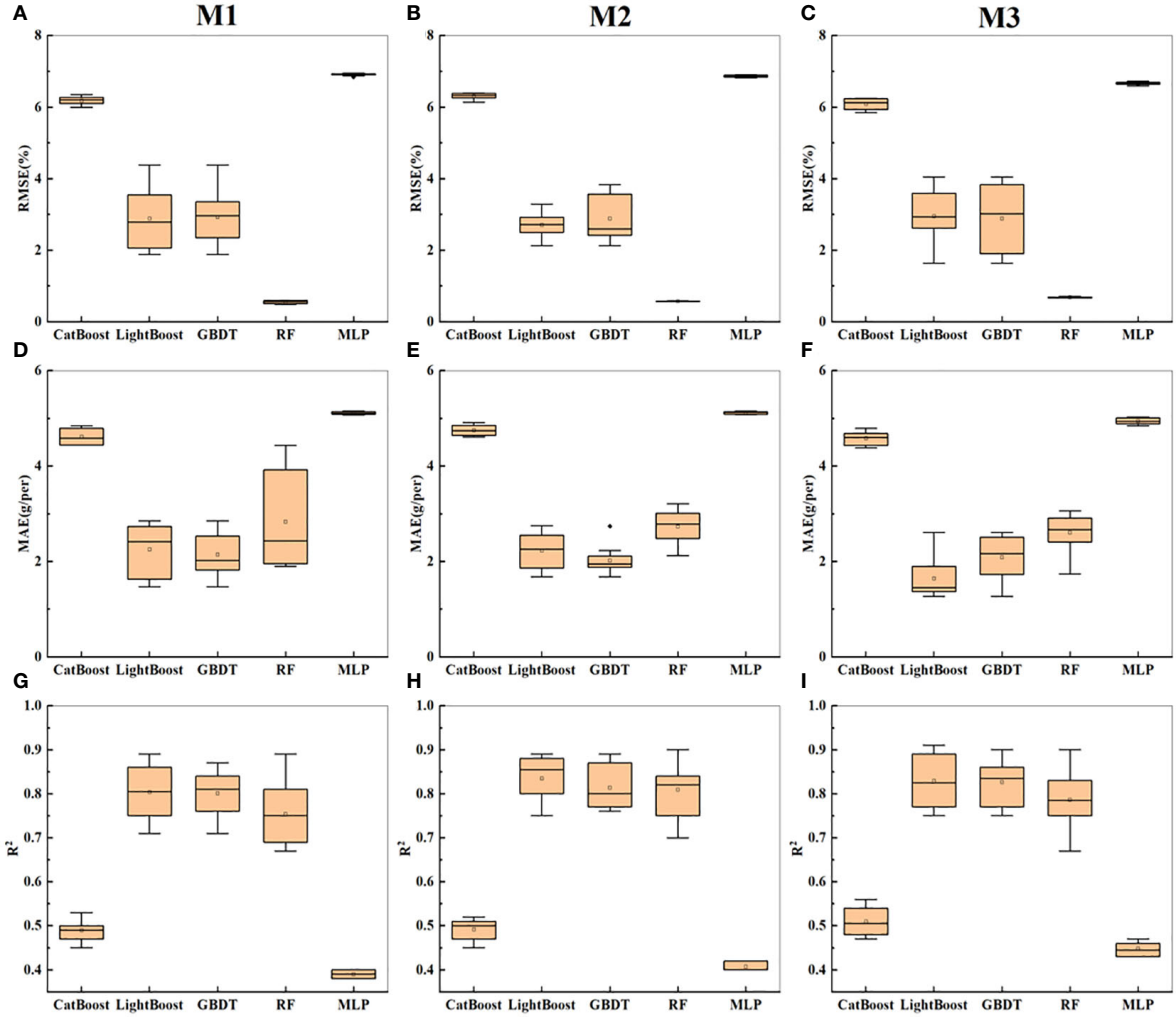
Presents the statistical results of yield prediction cross-validation based on top image parameters. **(A–C)** represent the RMSE of combinations M1-M3 across five machine learning models. **(D–F)** represent the MAE of combinations M1-M3 across five machine learning models. **(G–I)** represent the R^2^ of combinations M1-M3 across five machine learning models.

As shown in **Table 3**, the R^2^ value on the training set was generally greater than that on the test set, and the RMSE and MAE were generally lower than those on the test set. For M1, the GBDT and LightGBM models achieved the best prediction accuracy, with R^2^ values of 0.71 each. RF had an R^2^ value of 0.67, while CatBoost and MLP had the lowest prediction accuracies (R^2^ ≤ 0.45). For M2, the overall prediction accuracy of the five models was greater than that for M1 (except for CatBoost). However, the GBDT and LightGBM models still achieved the highest prediction accuracies, each with an R^2^ value of 0.76, while CatBoost and MLP had the lowest prediction accuracies (R^2^ ≤ 0.40), with the highest errors. For M3, the combination of textural and morphological parameters did not significantly improve the prediction accuracy of the five models. According to **Figures 7A–I**, the error order is m2 > m1 > m3, and the R^2^ value order is m3 > m2 > m1. With an increase in the richness of the indicators, the prediction accuracy increased, and the error decreased. The textural parameters led to better performance than the morphological parameters in estimating yield. **Figure 7** shows the statistical results of cross-validation for soybean yield prediction based on the top-image parameters. M3 still performed the best overall, with the highest prediction accuracy for the GBDT model. Overall, in predicting soybean yield based on the top-image parameters, the best predictive model was GBDT, which was consistent with the conclusion in Section 3.2.

**Table 3:** Prediction of yield based on top-image parameters.

		m1			m2			m3		
		RMSE (%)	MAE (g/per)	R^2^	RMSE (%)	MAE (g/per)	R^2^	RMSE (%)	MAE (g/per)	R^2^
CatBoost	Val	5.99	4.44	0.53	6.14	4.61	0.52	5.84	4.38	0.56
	Cal	6.35	4.84	0.45	6.39	4.91	0.45	6.25	4.79	0.47
LightBoost	Val	1.88	1.47	0.89	2.12	1.68	0.89	1.63	1.27	0.91
	Cal	4.38	2.85	0.71	3.29	2.75	0.75	4.05	2.61	0.75
GBDT	Val	1.88	1.47	0.87	2.12	1.68	0.89	1.63	1.27	0.90
	Cal	4.38	2.85	0.71	3.83	2.74	**0.76**	4.05	2.61	0.75
RF	Val	0.60	1.90	0.89	0.56	2.12	0.9	0.66	1.74	0.90
	Cal	0.49	4.43	0.67	0.58	3.21	0.70	0.71	3.06	0.67
MLP	Val	6.84	5.15	0.40	6.82	5.15	0.42	6.72	5.03	0.43
	Cal	6.95	5.07	0.38	6.90	5.08	0.40	6.59	4.84	0.47

Val for the validation set, Cal for the test set. The bold font represents the optimal predicted values.

### 3.4 Yield prediction based on side- and top-image parameters

**Figure 8:**
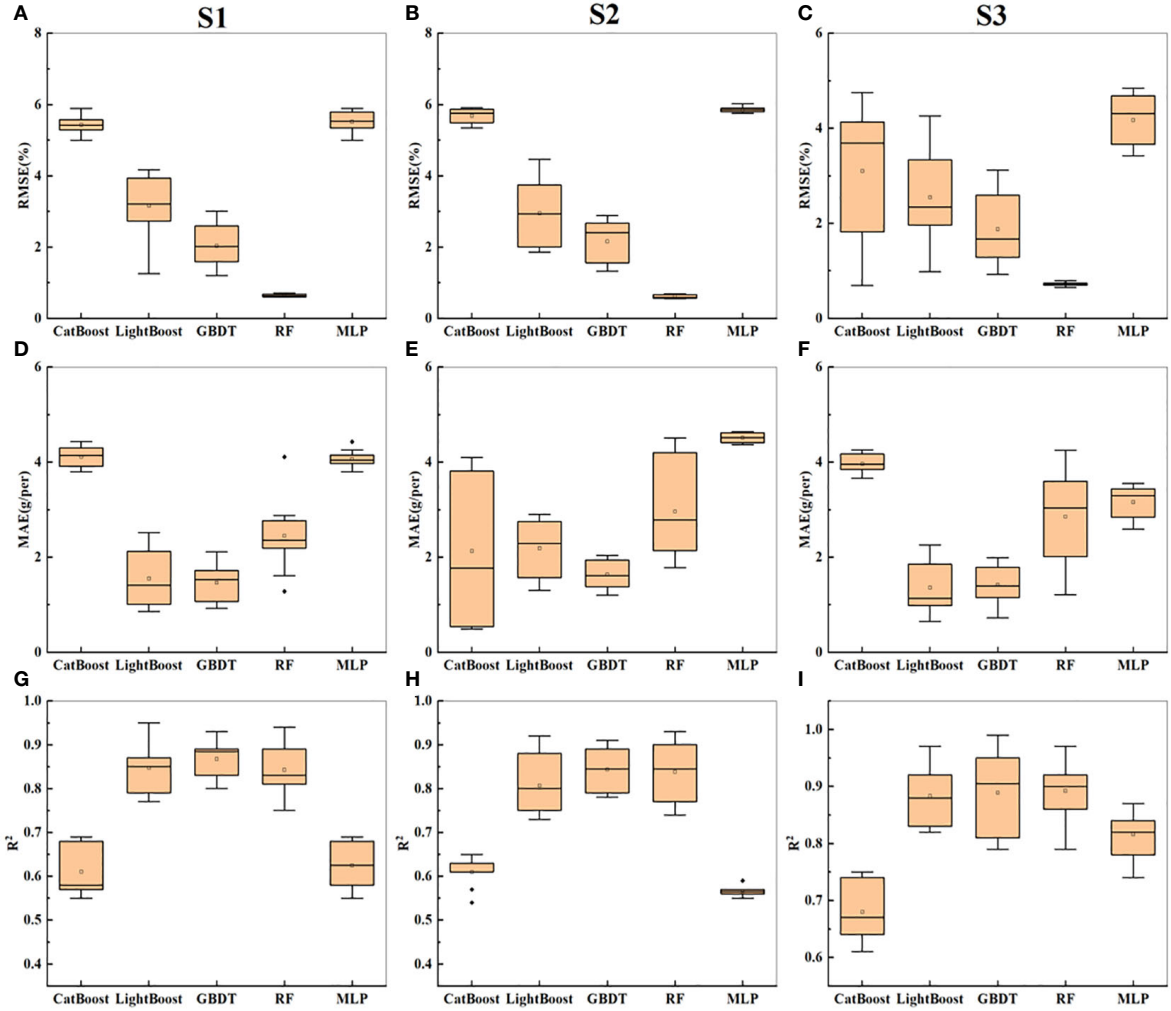
Presents the statistical results of yield prediction cross-validation based on top image parameters. **(A-C)** represent the RMSE of combinations S1-S3 across five machine learning models. **(D–F)** represent the MAE of combinations S1-S3 across five machine learning models. **(G–I)** represent the R^2^ of combinations S1-S3 across five machine learning models.

Building on the results from the previous sections, the side- and top-image parameters were combined to estimate the soybean yield. The three combinations of input variables were named S1-S3: S1 (m1 + M1), S2 (m2 + M2), and S3 (m3 + M3). The prediction accuracies of the CatBoost, RF, and MLP models improved compared to those presented in the previous sections. Notably, the MLP model demonstrated the most significant improvement, with higher prediction accuracy as the input indicators became more diverse, reaching a maximum of 0.74. The GBDT model also exhibited improved prediction accuracy and reduced errors compared to those in the previous sections, with RMSE = 3.12 and MAE = 1.99 on the test set. Moreover, the GBDT model consistently performed well. According to **Figures 8A–I**, the fusion of multiangle information contributed to reducing errors and enhancing the model prediction accuracy. In summary, the GBDT model is the optimal model for predicting soybean yield based on RGB image parameters. **Figure 8** shows the statistical results of cross-validation for soybean yield prediction based on the side- and top-image parameters. The fusion of multiangle information helps reduce errors and improve the model’s prediction accuracy.

### 3.5 Importance of input parameters

Based on the results presented above, GBDT emerged as the best predictive model, achieving the highest R^2^ value with the S3 combination while maintaining relatively low RMSE and MAE values. To further explore the contribution of each indicator and achieve high predictive accuracy with minimal input parameters, an analysis of the importance of each indicator with respect to the S3-GBDT combination was conducted.

**Figure 9:**
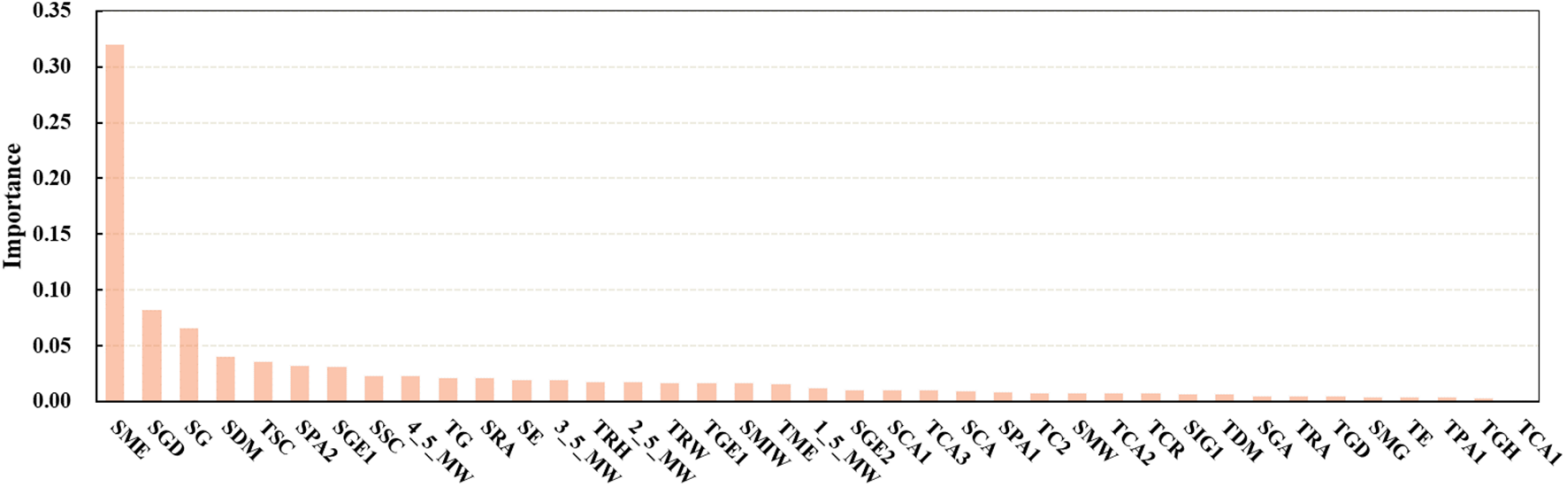
Importance of optimal input parameters.

As shown in **Figure 9**, the importance of the SME far exceeded that of the other indicators. This heightened importance could be attributed to its association with texture and photosynthesis (Heckmann et al., 2017). In addition to the SME, indicators such as SGD, SG, SDM, and TSC also exhibited considerable importance.

### 3.6 Selection of optimal input parameters

**Figure 10:**
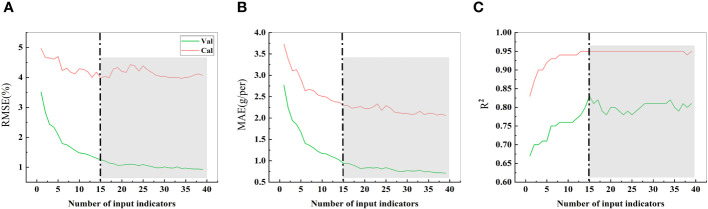
RMSE, MAE, and R^2^ values with different single seed weight input indices. **(A)** represent the RMSE. **(B)** represent the MAE. **(C)** represent the R^2^. The indicators are sequentially accumulated based on their importance. For instance, 1 item represents SME, 2 items represent SME + SGD, 3 items represent SME + SGD + SG, 4 items represent SME + SGD + SG + SDM, 5 items represent SME + SGD + SG + SDM + TSC, and so forth until all 39 indicators are included. The gray background indicates that, from the 15th item onward, the predictive performance of subsequent parameter combinations is comparable.

In the process of estimating crop yield based on remote sensing data, the selection of model variables is crucial (Ji et al., 2022). A comparison of the estimated soybean yield under the different numbers of input parameters (**Figures 10A–C**) revealed that the RMSE, MAE, and R^2^ values stabilized when the number of input parameters was equal to or greater than 15. To achieve high prediction accuracy with the most lightweight set of input parameters, we identified the 15 most common indicators (6 side textural indicators, 6 side morphological indicators, 1 top textural indicator, and 2 top morphological indicators) as the final selection of predictive parameters.

**Figure 11:**
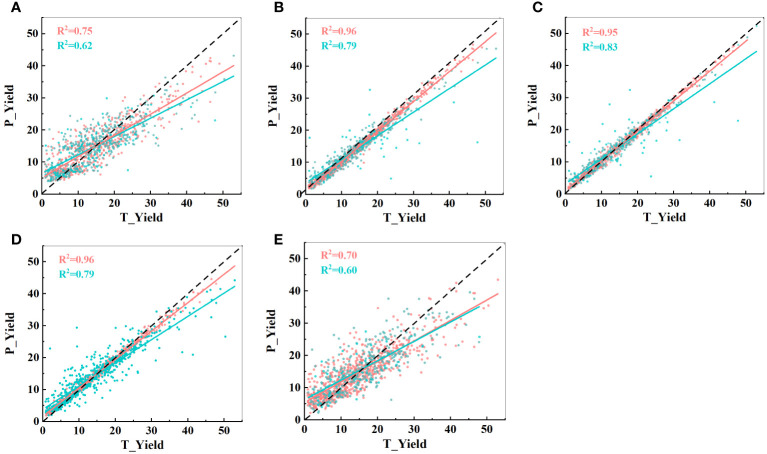
Predictive performance of the five models with 15 input indicators. **(A)** CatBoost model; **(B)** LightBoost model; **(C)** GBDT model; **(D)** RF model; and **(E)** MLP model. T_Yield represents the manually measured actual yield, and P_Yield represents the predicted value. The black dashed line represents a 1:1 relationship, where red indicates the training set and blue indicates the test set.

Using these 15 indicators as input parameters for the five models, the prediction results are shown in **Figure 11**. The prediction accuracy significantly decreased in the MLP model, while the other four models showed no significant changes. In fact, the GBDT model exhibited a slight improvement in R^2^ values. These results further emphasize that the effective selection of input indicators can greatly reduce the computational load while maintaining the prediction accuracy. Filtering out irrelevant information is advantageous for constructing a lightweight model.

According to **Table 4**, the broad-sense heritability of the 15 input parameters ranges from 0.68 to 0.84, with SME and SRA exhibiting the highest broad-sense heritability.

**Table 4:** The broad-sense heritability of the 15 input indicators.

Indicators	TRH	TSC	TG	2_5_MW	3_5_MW	4_5_MW	SRA	SSC	SPA2	SGD	SE	SG	SGE1	SME	SDM
*H^2^*	0.81	0.80	0.68	0.76	0.80	0.77	0.84	0.78	0.77	0.82	0.70	0.78	0.68	0.84	0.73

## 4 Discussion

### 4.1 Extraction of image parameters

There has been extensive research on the use of RGB cameras to extract image parameters for predicting crop yields. However, previous studies have often had relatively narrow shooting angles, mostly capturing images from the top, leading to significant information loss. Moreover, in previous research, the focus was primarily on morphological indicators (Ma et al., 2022). reported a significant correlation between vegetation indices extracted from RGB images and cotton yield, suggesting that selecting optimal parameter features for cotton yield monitoring is feasible. However, from the perspective of model accuracy, there is room for improvement. Therefore, there is a need to extract more textural features or other color information from RGB images to enhance the model accuracy. Research by (Bai et al., 2022) also demonstrated that textural information has high potential for yield estimation. Building on previous studies, in this study, not only were the shooting angles expanded from the top and side, but three major categories of image parameters were also extracted—morphology, color, and textural parameters. This approach significantly enriched the variety of image parameters, providing a broader selection for identifying the optimal image indicators in subsequent analyses.

### 4.2 Discussion on optimal input parameters

Indicator selection is a crucial step in machine learning. While algorithms determine the lower bounds of a model, input indicators set the upper bounds (Ma et al., 2022). The inclusion of a large amount of irrelevant input information can adversely affect model training, thereby impacting model accuracy. Therefore, a correlation analysis was initially conducted between all images and soybean yields, and only indicators with a Pearson correlation coefficient greater than 0.5 were obtained. This preliminary screening filtered out a significant amount of ineffective information.

After identifying GBDT as the optimal predictive model, further efforts were made to streamline the input indicators. Based on their importance, indicators were incrementally added to the model. As shown in **Figure 10**, using 15 important parameters as input led to the desired results. With an increase in the input parameters, the RMSE, MAE, and R^2^ values no longer exhibited significant changes, which might be due to the substantial collinearity among the input parameters, as the information coverage did not significantly improve with additional input indicators. Early analysis of input variables to reduce autocorrelation effects appears to have positive effects on model prediction (Han et al., 2019).

Moreover, an in-depth analysis of the final selection of 15 indicators, as shown in **Figure 9**, revealed that the importance of SMEs was the highest, exceeding 30%. Previous research has indicated an association between textural parameters and plant photosynthesis (Heckmann et al., 2017). Our experimental design included two planting modes, soybean monoculture and strip intercropping, which magnified the impact of light conditions on soybean yield. Consequently, the importance of textural parameters became more prominent, further confirming our hypothesis. Additionally, the number of selected sideview image indicators far exceeded that of the top-view indicators. This dataset included 12 sideview image indicators (6 textural indicators and 6 morphological indicators) and 3 top-view image indicators (1 textural indicator and 2 morphological indicators). From **Tables 1** and **2**, it is evident that predictions based on sideview images were better than those based on top-view images, and the selected indicators explain this predictive performance.

### 4.3 Discussion on the performance of the five models

As a branch of artificial intelligence, machine learning technology has found widespread application due to its remarkable ability to integrate complex and dynamic biological knowledge with large-scale omics data (Shahhosseini et al., 2020; Van Klompenburg et al., 2020; Paudel et al., 2021). Machine learning technologies enable the construction of various prediction models and decision algorithms (Yan and Wang, 2022). As evident from **Tables 2**, **3**, and **5**, the GBDT model achieved superior predictive accuracy compared to that of the other four models, consistently exhibiting the same pattern in terms of prediction errors. Therefore, the GBDT model is the optimal model for soybean yield estimation. The inherent advantage of GBDT, which is composed of numerous decision trees, lies in its continuous feature selection and partitioning process, which enhances its fitting capacity to the data (Zhang et al., 2023). GBDT achieves sample data classification by progressively reducing the residuals generated during the training process, significantly improving the data-fitting capabilities (Zhang et al., 2023).

**Table 5:** Prediction of yield based on side- and top-image parameters.

		S1			S2			S3		
		RMSE (%)	MAE (g/per)	R^2^	RMSE (%)	MAE (g/per)	R^2^	RMSE (%)	MAE (g/per)	R^2^
CatBoost	Val	5.00	3.80	0.69	5.34	4.10	0.65	4.75	3.66	0.75
	Cal	5.89	4.43	0.55	5.91	0.49	0.54	0.69	4.26	0.61
LightBoost	Val	1.25	0.86	0.95	1.86	1.30	0.92	0.98	0.65	0.99
	Cal	4.17	2.52	0.77	4.46	2.90	0.73	4.26	2.26	0.79
GBDT	Val	1.20	0.92	0.93	1.32	1.20	0.91	0.92	0.72	0.97
	Cal	3.01	2.11	0.8	2.88	2.04	0.78	3.12	1.99	**0.82**
RF	Val	0.71	1.28	0.94	0.69	1.78	0.93	0.79	1.21	0.97
	Cal	0.60	4.11	0.75	0.55	4.51	0.74	0.65	4.25	0.79
MLP	Val	5.00	3.80	0.69	6.02	4.64	0.55	3.42	2.59	0.87
	Cal	5.89	4.43	0.55	5.75	4.37	0.59	4.84	3.55	0.74

Val for the validation set, Cal for the test set. The bold font represents the optimal predicted values.

The overall prediction performance of the MLP model is relatively poor. This is attributed to the model’s network structure, which includes multiple hidden layers, and each “neuron” is connected to all nodes in the preceding layer. This structure results in many parameters, making training challenging. However, with sufficient computational power and training data, the MLP performance can be significantly enhanced. This finding aligns with the results of our study, where an increase in the input parameters led to a noticeable improvement in the MLP prediction performance, reaching a maximum R^2^ value of 0.74.

In this study, there are likely two reasons for the low predictive accuracy of the CatBoost model. First, due to the soybeans being grown in the field and influenced by field conditions, the growth status of each soybean plant is not very standardized (it may tilt, bend, break at the top, etc.; see **Supplementary Figure S2**), leading to many outliers. Although this study used the outlier test function in the car package to remove outliers, there is still a possibility of incomplete removal, which affects the predictive accuracy of the CatBoost model. Second, this study referred to the methods of predecessors and conducted preliminary screening of input parameters using absolute correlation (Yu and Liu, 2003; Cui et al., 2010; Ma et al., 2022; Li et al., 2023). However, it is possible that setting the screening threshold too low (Cor > 0.5) retained some indicators with weaker relationships, which had a certain impact on the predictive accuracy of the model. Therefore, in future research, more screening criteria should be added on the basis of correlations to enhance the initial screening of indicators. In the context of machine learning, heritability may not be a direct factor influencing algorithm selection, but it does affect the nature of the data and the structure of the feature space, thereby indirectly influencing the selection and performance of machine learning algorithms. In this study, the broad-sense heritability of soybean yield was 0.621. Therefore, when selecting machine learning algorithms, features with complex genetic mechanisms may have more diverse and nonlinear distributions. Algorithms capable of handling nonlinear relationships and complex patterns, such as support vector machines or decision trees, may be more suitable.

In general, as the richness of the indicators increases, the prediction accuracy improves, and the errors decrease. When using single-angle indicators as the input parameters, sideview indicators lead to significantly better performance than top-view indicators. When single-type indicators are used as the input parameters, the morphological parameters lead to better performance than the textural parameters from the sideview, while the textural parameters lead to better performance than the morphological parameters from the top view. We speculate that this phenomenon is due to the abundance of effective morphological parameters extracted from sideview images, whereas the top view provides a more intuitive view of young and tender soybean leaves. Therefore, sideview morphological parameters and top-view textural parameters are more critical for estimating soybean single-plant yield.

### 4.4 Future directions

In this study, five machine learning algorithms were employed to estimate soybean yield. The results indicate that RGB images can be used to accurately estimate soybean yield. This research contributes to the identification of a high-throughput and nondestructive method for estimating soybean yield, accelerating the screening of germplasm and breeding materials.

Despite extracting as many indicators as possible in this study to estimate the final yield parameters, the information inherently contained in RGB images is limited, leaving room for further improvement in terms of the estimation accuracy. However, this study, which was based on data from 240 soybean varieties, two treatments, and two years of field data, benefitted from a large dataset, making our conclusions more applicable to real-world scenarios and highly credible. Future research could consider adding sensor types and enriching phenotypic parameters, thereby enhancing the prediction accuracy and reducing errors. Additionally, due to limited throughput and substantial workload, only images from the R6 stage were collected for analysis in this study. Subsequent research could involve image collection throughout the entire growth period to determine the earliest stage for accurate soybean yield estimation, further shortening the breeding process.

Finally, it is worth noting that selecting the best predictive model requires considering multiple factors comprehensively. Key factors include data characteristics such as scale, number and type of features, distribution, problem nature such as regression or classification, order or unordering, model characteristics such as interpretability, training speed, overfitting and underfitting, and resource constraints such as computation and time. For example, when dealing with many features, tree-based algorithms (such as GBDT and RF) may perform well because they can effectively handle high-dimensional data. For features with complex relationships, neural networks may be more effective. For regression problems, linear regression, decision tree regression, or GBDT may be good choices; for classification problems, logistic regression, support vector machine (SVM), RF, or deep neural networks may be more suitable. Additionally, it is important to evaluate model performance and select appropriate metrics through cross-validation. Therefore, there is no universally best model, and it is necessary to try multiple models and optimize them based on specific circumstances.

## 5 Conclusions

This study concludes with two main findings: (1) The optimal input parameters were determined to be SME, SGD, SG, SDM, TSC, SPA2, SGE1, SSC, 4/5MW, TG, SRRA, SE, 3/5MW, TRH, and 2/5MW, totaling 15 indicators. (2) Among the five machine learning models, GBDT emerged as the best model for predicting soybean yield, achieving a maximum accuracy of 0.82. Therefore, this combination of parameters extracted from RGB images and machine learning has significant potential for estimating soybean yield parameters.

## Data availability statement

The raw data supporting the conclusions of this article will be made available by the authors, without undue reservation.

## Author contributions

XL: Conceptualization, Writing – original draft, Writing – review & editing, Data curation, Visualization. MC: Data curation, Writing – original draft. SH: Conceptualization, Writing – review & editing. XX: Visualization, Writing – original draft. LH: Validation, Writing – review & editing. LW: Validation, Writing – review & editing. YG: Software, Writing – review & editing. FT: Software, Writing – review & editing. TG: Software, Writing – review & editing. WW: Supervision, Writing – review & editing. MX: Supervision, Writing – review & editing. CL: Supervision, Writing – review & editing. LY: Resources, Writing – review & editing. WL: Funding acquisition, Writing – review & editing. WY: Writing – review & editing, Resources.
