# Supplementary material for: Estimation of soybean yield based on high-throughput phenotyping and machine learning
Source: Front Plant Sci. 2024 Jun 6;15:1395760. doi: 10.3389/fpls.2024.1395760 (PMC11187272; doi:10.3389/fpls.2024.1395760)
Supplement: Supplementary file 1 [file Presentation_1.zip › Supplementary Material/Appendix 2:Definition of the image metrics.DOCX]

**Definition of traits and calculation formulas**

**Color parameters**

- Blue Mean: In the RGB three channels, only the value of the blue channel is retained, and the mean of the blue channel in the pixel where the soybean plant is located is calculated.
- Green Mean: In the RGB three channels, only the value of the green channel is retained, and the mean of the green channel in the pixel where the soybean plant is located is calculated.
- Red Mean: In the RGB three channels, only the value of the red channel is retained and the mean of the red channel in the pixel where the soybean plant is located is calculated.
- Blue-green ratio: The ratio of the blue mean to the green mean.
- Blue-red ratio: The ratio of the blue mean to the red mean.
- Green-to-red ratio: The ratio of the green mean to the red mean.

**Pattern parameters**

- Perimeter: Calculated from the foreground pixels of the outermost circle of the soybean plant.
- Projection area: Calculated from the number of pixels in the foreground of the soybean plant.
- Contour area: Calculated from the foreground pixels of the area enclosed by the outermost circle of the soybean plant.
- External rectangle width: that is, the maximum width of the soybean plant.
- External rectangle height: that is, the maximum height of the soybean plant.
- External circle radius: Calculated from the pixels of the outer circle radius of soybean plants.
- External rectangular area: that is, plant height * plant width.
- External circle area: calculated from the pixels of the area besieged by the outer circle of soybean plants.
- Shape rate compactness: Compactness is a measure of the shape characteristics of an area. Due to the different shape of the plane, the space of the same projection area reflects the compactness of its spatial distribution, and the calculation formula for the compactness of the shape rate is 1.273A/L2, A is the area, and L is the longest axis of the area.
- Roundness compactness: The roundness compactness is calculated as 4ΠA/P2, A is the area area, and P is the area circumference.
- External circle compactness: The compactness of the external circle is calculated as A/A', A is the area area, and A' is the minimum external circle area.
- Ratio of projected area to external rectangular area: The ratio of the projected area of a soybean plant to the area of its external rectangle.
- Ratio of height to width: The ratio of soybean plant height to width.
- 1/5 area ratio: calculated from the pixels of the area occupied by the plant in the height of 0 to 1/5 of the soybean plant image.
- 2/5 area ratio: calculated from the pixels of the area occupied by the plant in the height of 1/5 to 2/5 of the soybean plant image.
- 3/5 area ratio: calculated from the pixels of the area occupied by the plant in the height of 2/5 to 3/5 of the soybean plant image.
- 4/5 area ratio: calculated from the pixels of the area occupied by the plant in the height of 3/5 to 4/5 of the soybean plant image.
- 5/5 area ratio: calculated from the pixels of the area occupied by the plant in the height of 4/5 to 5/5 of the soybean plant image.
- 1/5 maximum width: Calculated from pixels at the widest point in the height of 0 to 1/5 of the soybean plant image.
- 2/5 maximum width: Calculated from pixels at the widest point in the height of 1/5 to 2/5 of the soybean plant image.
- 3/5 maximum width: Calculated from pixels at the widest point in the height of 2/5 to 3/5 of the soybean plant image.
- 4/5 maximum width: Calculated from pixels at the widest point in the height of 3/5 to 4/5 of the soybean plant image.
- 5/5 maximum width: Calculated from pixels at the widest point in the height of 4/5 to 5/5 of the soybean plant image.
- Average width: The average width.
- Crown height: The height of the widest part of the soybean plant.
- Crown height to plant height: the ratio of canopy height to plant height.
- Crown width: Calculated from the number of pixels at the widest point of the soybean plant.
- Minimum width: Calculated from the number of pixels in the narrowest part of the soybean plant.
- Convex envelope area: calculated from the number of pixels in the area besieged by the vertex of the soybean plant.
- Number of convex scales: The number of vertices.
