# Supplementary material for: Estimation of soybean yield based on high-throughput phenotyping and machine learning
Source: Front Plant Sci. 2024 Jun 6;15:1395760. doi: 10.3389/fpls.2024.1395760 (PMC11187272; doi:10.3389/fpls.2024.1395760)
Supplement: Supplementary file 2 [file DataSheet_1.docx]

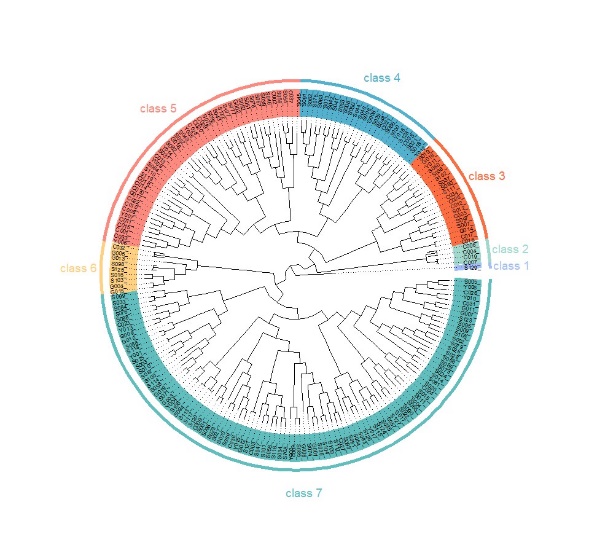


Figure S1. Cluster analysis of 240 germplasm resources


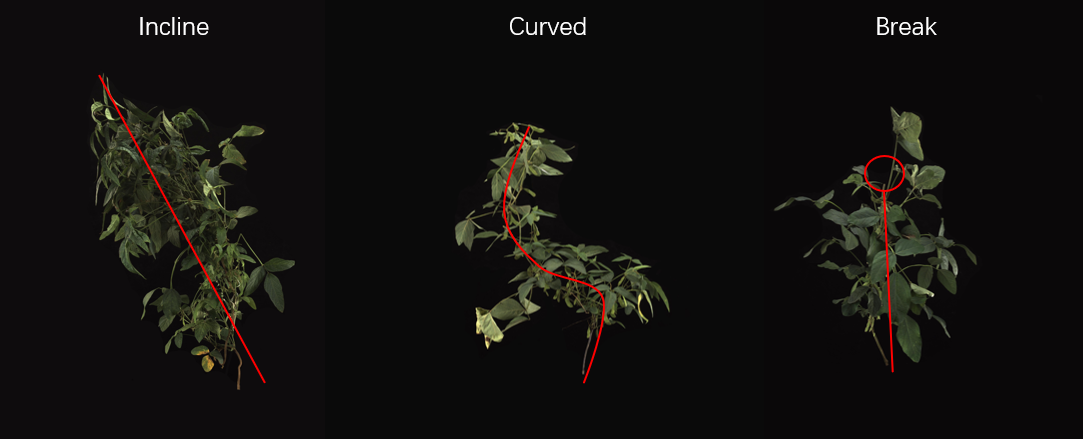


Figure S2. Diagram of extreme material field growth.
